# Integration of Complementary and Alternative Medicine into Family Practices in Germany: Results of a National Survey

**DOI:** 10.1093/ecam/nep019

**Published:** 2010-10-19

**Authors:** Stefanie Joos, Berthold Musselmann, Joachim Szecsenyi

**Affiliations:** ^1^Department of General Practice and Health Services Research, University Hospital Heidelberg, Voßstrasse 2, 69115 Heidelberg, Germany; ^2^Practice of Family Medicine, Academic Teaching Practice, University of Heidelberg, Hauptstrasse 120, 69168 Wiesloch, Germany

## Abstract

More than two-thirds of patients in Germany use complementary and alternative medicine (CAM) provided either by physicians or non-medical practitioners (“Heilpraktiker”). There is little information about the number of family physicians (FPs) providing CAM. Given the widespread public interest in the use of CAM, this study aimed to ascertain the use of and attitude toward CAM among FPs in Germany. A postal questionnaire developed based on qualitatively derived data was sent to 3000 randomly selected FPs in Germany. A reminder letter including a postcard (containing a single question about CAM use in practice and reasons for non-particpation in the survey) was sent to all FPs who had not returned the questionnaire. Of the 3000 FPs, 1027 (34%) returned the questionnaire and 444 (15%) returned the postcard. Altogether, 886 of the 1471 responding FPs (60%) reported using CAM in their practice. A positive attitude toward CAM was indicated by 503 FPs (55%), a rather negative attitude by 127 FPs (14%). Chirotherapy, relaxation and neural therapy were rated as most beneficial CAM therapies by FPs, whereas neural therapy, phytotherapy and acupuncture were the most commonly used therapies in German family practices. This survey clearly demonstrates that CAM is highly valued by many FPs and is already making a substantial contribution to first-contact primary care in Germany. Therefore, education and research about CAM should be increased. Furthermore, with the provision of CAM by FPs, the role of non-medical CAM practitioners within the German healthcare system is to be questioned.

## 1. Background

The growing popularity of complementary and alternative medicine (CAM) is associated with an ongoing debate of integrating such therapies into mainstream healthcare [1, 2]. The extent to which CAM is practiced by physicians and/or non-medical therapists considerably differs among countries [3–12]. Recent data suggest that patients believe that there is an increased need for family physicians involvement in providing and supervising CAM treatments [13]. In the United States, CAM is widely used by the general population [14] and total out-of-pocket expenditures for CAM have been conservatively estimated to be $27 billion [15]. Also, the acceptance of CAM among physicians has increased in the last years [16, 17]. However, most family physicians (FPs) in the United States are not being trained in CAM. In a survey of FPs, 76% said their patients use CAM, and the overwhelming majority (84%) thought they needed to learn more about CAM to adequately address patient concerns [17]. As a first step in this direction, the Group on Alternative Medicine of the Society of Teachers of Family Medicine has developed curriculum guidelines for programs wishing to include formal training in CAM in residency training [18]. Thereupon, family medicine residency programs incorporating CAM into their curriculum have been developed with financial support of the National Center for Complementary and Alternative Medicine in the United States [19, 20].

In Germany, the overall percentage of individuals with experience in CAM increased from 52% in 1970 to 73% in 2002 [21]. Specific CAM disciplines (naturopathy, chiropractic, homeopathy, physical therapy, balneology & medical climatology, acupuncture) are accredited by the German Federal Medical Chamber. FPs can obtain additional CAM qualification after a theoretical and practical training. At the end of 2006, a number of 47 193 CAM qualifications were registered among all 407 000 German physicians in Germany [22, 23]. In addition, many FPs are providing CAM in their daily practice without having any CAM certification. However, to date there is little evidence about how many FPs provide CAM in day-to-day practice.

In Germany, only a small part of CAM is covered by the statutory health insurance (SHI), namely physiotherapy, chiropractic, classic naturopathy, homeopathy to a very small extent and, newly, acupuncture, in patients with knee pain and lumbar pain. A physician must hold the corresponding CAM qualification for reimbursement by SHI. All remaining CAM therapies are not covered by SHI but have to be paid by the patients themselves or may be reimbursed by private health insurances. Moreover, since the beginning of January 2004, all homeopathic and phytotherapeutic drugs (with the exception of mistletoe, St John's wort, psyllium and ginkgo) are non-reimbursable by the SHI [24].

Apart from physicians there are non-medical, state-licensed practitioners in Germany, so-called “Heilpraktiker”, providing a great variety of CAM therapies. “Heilpraktiker” is not an occupation requiring formal training neither for basic medical knowledge nor for CAM. However, a “Heilpraktiker” has to pass an exam on basic medical knowledge and skills at a local public health office to obtain a state license [25]. Corresponding to the growing demand for CAM in the public, the number of “Heilpraktiker” increased from 9000 in the year 1993 to nearly 20 000 today. For some patients, the Heilpraktiker already replaces the family doctor.

This is the first national survey we are aware of to ascertain the use of CAM by FPs and their attitudes toward specific CAM disciplines in Germany. Furthermore, questions with regard to common conditions for CAM, attitudes toward Heilpraktiker, education and research about CAM were asked during the survey.

## 2. Methods

**Figure 1:**
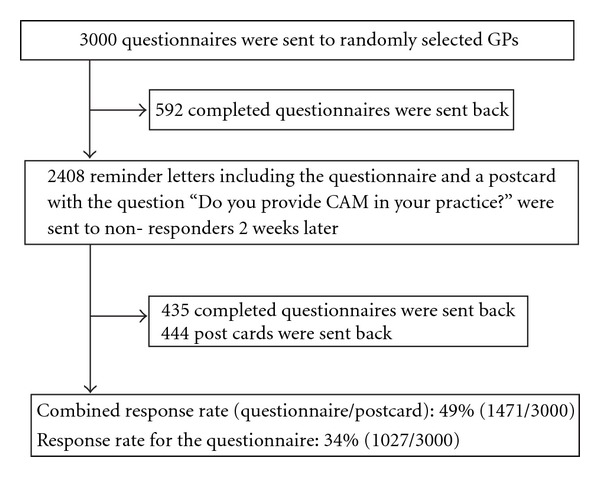
Flow chart.

The presented study was designed as a cross-sectional survey with a nationwide random sample of FPs (included were “Fachärzte für Allgemeinmedizin” and “praktische Ärzte”). Addresses were obtained from the databases of the Regional Associations of SHI-Accredited Physicians. Physicians were invited by letter, which was spiced up with a tea bag for relaxation (provided by the Ostfriesische Tee Gesellschaft/Laurens Spethmann GmbH & Co, Seevetal, Germany). In March 2007, questionnaires were sent to 3000 randomly selected FPs (Figure 1). A reminder letter, including the questionnaire once more was sent to non-responders 2 weeks later. A postcard was attached to the reminder letter, which included the following questions: “Do you provide CAM in your practice?” (yes/no) and “Why do you refuse to complete the questionnaire?” (no time/do not practice as a FP/in principle not taking part in surveys/*miscellaneous*). FPs were asked either to complete the questionnaire or at least to send back the postcard.

### 2.1. Questionnaire

The questionnaire was developed based on a preceding focus group study [23]. Altogether, the questionnaire comprised 50 questions about the following topics: rating of CAM, reality of (CAM) care, philosophy of care, job satisfaction and demographics. Within this article, results of the topics “rating of CAM”, “reality of (CAM) care” and demographics are presented. For the purpose of this study, CAM was defined as “all diagnostic and therapeutic procedures not ranking among conventional medicine (“Schulmedizin”)”. In this definition, classical naturopathic methods (hydrotherapy, phytotherapy, kinesiotherapy, dietetics, physical and regulative therapy), other traditional healing systems, as well as less well-known and used therapies such as bioresonance therapy and autohemotherapy were included. For two questions regarding use and attitude toward CAM, a predefined list of CAM disciplines was given. This list included the following methods: acupuncture, anthroposophic medicine, autohaematotherapy, relaxation, homeopathy, regulative therapy (“Ordnungstherapie”), manual therapy/chirotherapy/osteopathy, neural therapy, orthomolecular therapy and phytotherapy. The term neural therapy is used for the therapeutical approach of treating medical problems by injecting local anesthetics symptomatically in triggerpoints or according to Huneke into so-called “interfering field”.

Most items were measured on 5-point-Likert scale. To obtain better overview, responses are summarized within three categories in the result section. Furthermore, within the questionnaire, statements about specific aspects of CAM were given which should be rated by the FPs on 5-point-Likert scales (1 = strongly diasagree, 3 = neutral, 5 = strongly agree). The questionnaire was tested among 20 FPs and revised according to the given notes and suggestions of improvement. The original questionnaire (German language) can be requested from the corresponding author.

### 2.2. Statistics

For statistical calculations we used SPSS 15.0 software. In cases of less than 3% missing values, percentages are given as valid percentages, which means that they are summed up to 100%. Otherwise, the numbers of missing values are stated explicitly.

To check for representativeness, demographic data of our sample are compared with data of the German Physician sample 2006 produced by the National Association of SHI-Accredited Physicians [22] and the sample of the Commonwealth Fund Survey 2006 [26].

## 3. Results

Of the 3000 FPs, 1027 returned the questionnaire and 444 FPs returned the postcard. Thus, for the key question “Do you provide CAM in your practice” the combined response rate (questionnaire + postcard) was 49%. For the remaining questions, the response rate was 34% (Figure 1). The 444 FPs returning only postcards indicated the following reasons for not completing the whole questionnaire: 215 FPs indicated “no time”, 141 indicated “in principle not taking part in surveys”, 38 did not like the questionnaire, and 13 did not practice (anymore) as FP.

In Table 1, demographic data of our sample is displayed. FPs in our sample had various CAM certifications headed by acupuncture certification. The mean age of respondents was 51 years with ages ranging from 30 to 71 years. Mean duration of years in practice was 15 years ranging from 1 to 36 years. Mean percentage of privately insured patients per practice was 12%.

### 3.1. Use of CAM in Practice

Of the 1027 FPs, 737 (72%) responded to the question “Do you use CAM in your every-day practice?” with “yes”, 141 (14%) responded “no” and 149 (14%) did not answer the question. Of the 444 FPs returning only the postcard, 149 (34%) indicated that they use CAM in their practices, whereas 198 (45%) negated this question and 97 (22%) FPs did not answer it at all. Thus, altogether 886 FPs of 1471 FPs (60%) indicated to use CAM in their practice.

**Figure 2:**
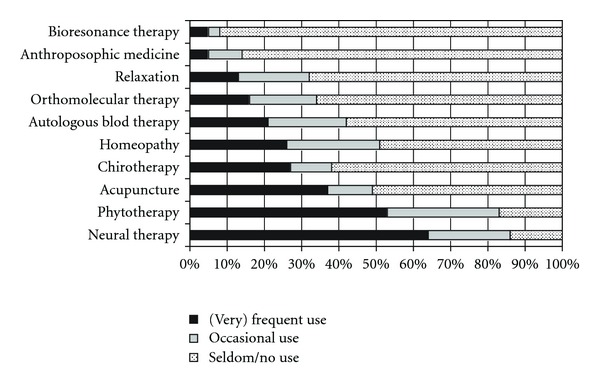
Use of specific CAM therapies in practice in the last 12 months.

Figure 2 displays the frequency to which the different CAM disciplines had been used by the FPs in the previous 12 months. Neural therapy was the CAM therapy most frequently used with 565 out of 872 FPs indicating a (very) frequent use in practice. It was followed by phytotherapy (*n* = 459/873) and acupuncture (*n* = 316/858). On average, FPs applicate CAM in 25.5% of their patients (SD 25.1; 15% missing items). Only 64 out of 737 FPs stated problems for a combination of CAM with conventional therapy.

### 3.2. Attitudes toward CAM and Rated Benefit of Specific CAM Therapies

**Figure 3:**
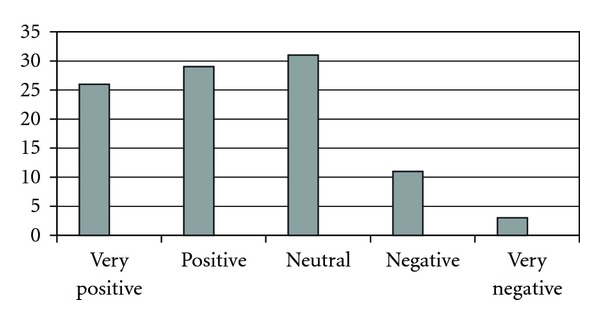
Overall attitude toward CAM (given as percentages of FPs).

**Figure 4:**
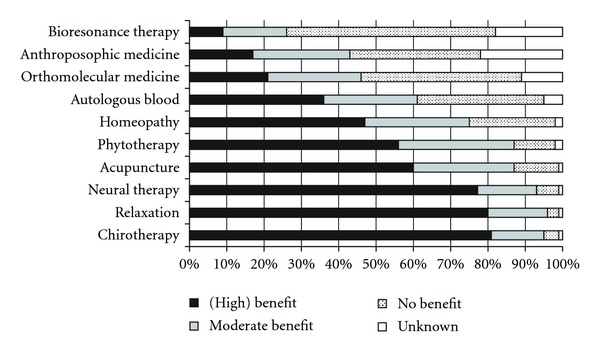
Benefit rating of specific CAM therapies.

The question concerning the overall attitude toward CAM was responded by 910 of the 1027 FPs (87%). Of these, 503 FPs indicated a “positive” or “very positive” attitude toward CAM, whereas 127 indicated a “negative” or “very negative” attitude (Figure 3). Chirotherapy was assessed as the most beneficial CAM discipline (with 818 out of 1016 FPs indicating a [high] benefit), followed by relaxation (*n* = 810/1013) and neural therapy (*n* = 790/1017) (Figure 4).

Of the statements, which were given in the questionnaire to be rated by FPs, most agreed was the statement that FPs should have a basal education about the most important CAM disciplines (73%), followed by the statement that research in CAM should be increased (68%). Most disagreed was the statement to refer a patient to a Heilpraktiker (Table 2). More than half of the FPs claimed for more control regarding patient care by Heilpraktiker.

### 3.3. Conditions Treated with CAM

**Figure 5:**
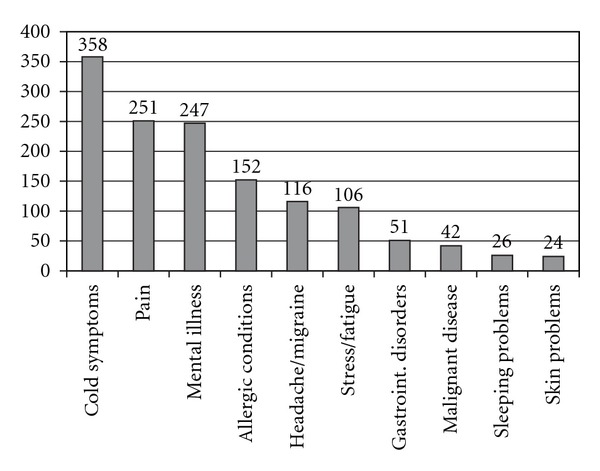
Type and number of mentioned conditions treated with CAM indicated by FPs.

Figure 5 displays type and number of mentioned conditions treated with CAM. The most frequent conditions for CAM indicated by FPs as free-text item were cold symptoms (indicated by 358 FPs) followed by pain (including musculoskeletal pain conditions) indicated by 251 FPs and mental illness indicated by 247 FPs (Figure 5).

## 4. Discussion

Given the widespread public interest in CAM, the aim of this study was to ascertain the use of and attitude toward CAM among FPs. To our knowledge, this is the first nationwide study supplying quantitative data regarding attitudes toward and use of CAM among FPs in Germany.

### 4.1. Use of CAM in Practice

In our study, 60% of the responding FPs indicated to provide CAM in practice, which is the highest percentage of CAM provision in comparison with other countries with percentages ranging between 13% and around 38% [4–10]. This high percentage is in accordance with a small non-representative survey of Himmel et al. in 1993 [11] and a subgroup analysis by Haltenhof et al. in 1995, revealing rates of 85% and 56%, respectively, for the provision of CAM by German FPs [12]. However, both studies are older than 10 years and hampered by methodological problems such as small sample sizes and convenience sampling.

Our study provides information about the most commonly used CAM disciplines in family practice, which are neural therapy and phytotherapy provided by 50–70% of FPs. Both therapies have a long tradition in German-speaking countries. Therefore, it is not surprising to find similar results in a Swiss survey [28], but lower rates in other countries [7, 8, 10, 29]. (Most surveys did not even ask for the use of “neural therapy”.) There is a robust evidence-base for several herbal drugs such as St. Johns wort for depression or Echinacea for common cold [30, 31]. For some commonly used herbal drugs such as milk thistle or Passiflora, the evidence is contradictory or insufficient to draw conclusions [32, 33]. Regarding neural therapy, there are data proving the efficacy of the injection of local anaesthetics into trigger points, for example, in neck disorders [34]. However, there is no proper randomized controlled study about neural therapy according to Huneke that is injecting local anaesthetics into so-called “interfering fields” [35].

Our study also demonstrates that more than one-third of FPs use acupuncture. The prevalent use of acupuncture, on the one side, may reflect the strong evidence-base of acupuncture particularly in pain disorders [36, 37] and the inclusion of acupuncture in guidelines [38, 39]. On the other side, prevalent acupuncture use in Germany can be explained by reimbursement of acupuncture for back pain and osteoarthritis by the SHI.

Therapies underpinned by little or no evidence, such as autologous blood therapy, or orthomolecular therapy, were provided by a lesser number of FPs. Sustained use of such CAM therapies may be explained by positive experiences of the individual FP and/or may just reflect a “pragmatic” therapeutic approach of the doctors. These therapies warrant further research to discover potential therapeutic effects but also to look for side effects.

### 4.2. Attitudes toward CAM and Perceived Benefit of Specific CAM Therapies

Only a few studies have included an “overall-CAM-attitude question”. Schmidt et al. investigated whether there is a difference in FPs' attitudes toward CAM in the United Kingdom and in Germany and found a (non-significant) more positive attitude in German FPs [40]. In a recently published Turkish survey, 51% of the requested FPs indicated to believe in the efficiency of CAM, whereas 38% did not [41].

In our study, the majority of FPs had a positive attitude toward CAM. One aim of this study was to assess generalized statements concerning CAM. This was to obtain an idea of the current general attitude in the “family medicine community” toward CAM. This, although having in mind that this atitude is not exclusively influenced by scientific evidence but also by political, social and economic factors.

In our study, chirotherapy and relaxation techniques were the most highly valued CAM therapies with more than 80% of FPs indicating a (high) benefit. Chirotherapy is supported by a robust evidence-base at least for acute low back pain and pain disorders of the skeletal system [42, 43]. Also for relaxation techniques such as yoga, there is evidence from several randomized controlled studies about potentially beneficial effects [44, 45]. However, only a minority of FPs provides these two CAM disciplines in practice. This might be due to the fact that many FPs have no formal training in these disciplines and, therefore, may refer their patients. Concerning relaxation it is supposed that patients use courses offered by gyms, sports clubs or other communal institutions.

More than 50% of the responding FPs in our survey thought that patients would (highly) benefit from acupuncture. This is in accordance with survey from other countries where acupuncture takes a “top position” among CAM disciplines [5, 8, 9, 11]. Between 30 and 50% of the FPs have a positive attitude toward homeopathy and autologous blood therapy, respectively, which is intriguing, considering the fact that both therapies lack a substantial evidence base.

Opinions whether CAM should be provided by non-medical practitioners or solely by physicians are not consistent among FPs. About 30% of the FPs would suggest a patient to go to a Heilpraktiker, but nearly 50% disagreed. About 60% of the FPs want more control concerning patient-care with CAM provided by Heilpraktiker. From our preceding qualitative study we know that FPs feel pressurized by more and more quality control in their day-to-day practice [23]. This is in contrast with lacking standards for of Heilpraktiker. Therefore, the claim for more quality control concerning the care provided by Heilpraktiker is understandable.

“Stiftung Warentest”, a foundation established by the German Bundestag with the aim of providing independent and objective support for consumers, recently assessed the quality of consultations in 40 Heilpraktiker by means of test-patients. The authors concluded that Heilpraktiker might have their strengths, for example, in service, structure of practice, and providing enough time and a pleasant practice atmosphere for patients. However, the authors raised serious concerns in particular regarding the quality of medical-history-taking (e.g., medication, conventional treatment), patient information (e.g., about the intended treatment including costs), documentation of the consultation, and education standards [46]. Indeed, Heilpraktiker are allowed to applicate injections, for instance, of homeopathic remedies, and other invasive procedures, without proving a formal education. Heilpraktiker still seem to have an exceptional role among healthcare provider in Germany. This may complicate progress concerning quality and research issues in CAM.

### 4.3. Condition for CAM Use

The most frequent conditions for CAM use were cold symptoms, pain (including musculoskeletal), and mental illnesses. Other surveys among FPs rarely asked for the conditions treated with CAM. However, studies investigating reasons for healthcare utilization underpin the significance of those three conditions in general practice [47, 48].

For the therapeutical management of cold symptoms and mental illnesses in primary care, herbal drugs such as Echinacea, Pelargonium sidoides or St. Johns wort play an important role in Germany [30, 31, 49]. For pain conditions of the musculoskeletal system, acupuncture and neural therapy may be the most commonly applied CAM disciplines.

### 4.4. Strengths and Limitations of the Study

A main strength of this study is the nationwide random sample. A further strength is the use of a questionnaire developed based on qualitative data [23] assuring that included questions refer to issues with practical relevance.

There was a response rate of nearly 50% for the key question and 34% for the remaining questions, which is acceptable compared to international studies surveying the provision of CAM among FPs [16, 17, 41]. The motivation of German doctors to participate in surveys of this type is generally low with rates between 15% and 30% [27, 50]. Therefore, the response rate in the presented study has exceeded expectations of the authors and may be seen as a result of a well-developed questionnaire. Nevertheless, the response rate leaves room for bias and may limit the extent to which these findings are representative for all German FPs. It may be possible that the proportions of CAM providing FPs may be overestimated. Since it was an pseudonymous survey, there is no information about non-responding FPs. However, based on the reasons for non-participation in the survey, which FPs had indicated on the postcards, it can be concluded that the reasons were mostly CAM-independent.

Moreover, comparisons with available data aiming at validating work parameters and demographic parameters at least confirm that respondents were representative in basic respects such as gender, age, and location of practice [22, 26, 50]. Also, the number of FPs holding a CAM qualification was only slightly higher in our sample compared with the German Physician Sample of 2006 [22]. Moreover, the indicated statements for not participating in the survey (48% “no time” and 32% “in principle not taking part in surveys”) do not argue for substantial bias. Supposing the case that all non-responders would not provide CAM would still result in a substantial rate of 30% (886/3000) CAM-providing FPs in Germany.

### 4.5. Implications for the Future

According to preceding qualitative research, patients believe a combined approach of CAM and conventional medicine is better than either alone. Patients wish to discuss CAM use with well informed FPs [51]. Hence, the high rate of CAM-providing FPs in Germany compared with other Western countries can be regarded as a beneficial situation for patients.

However, there is some homework Germany has to do: all CAM therapies with a proper evidence base should be reimbursed by the SHI. First steps in this direction are taken by SHIs offering the choice of specific CAM rates.

Furthermore, the role of Heilpraktiker within the German healthcare system urgently has to be reconsidered. Crucial points are the intransparency of education and of provided care and, alongside, the reimbursement of their services by private insurances or even SHI (within specific CAM rates). Standards about education and quality control should be introduced by healthcare policy. Furthermore, beliefs and existing practices of Heilpraktiker should be assessed, for example, by focus groups or in-depth interviews [52] to explore the chances for collaboration between FPs and Heilpraktiker.

Our study revealed a clear vote among FPs for more research and education in CAM, which is in accordance with the international literature [16, 17, 53]. Considering the relevance CAM has in the public, it must be claimed for state-funded research and education programmes such as the National Center for Complementary and Alternative Medicine in the United States and other initiatives [19]. In Germany, CAM has been integrated in undergraduate education in 2003 with the aim that students obtain a basic theoretical knowledge about frequently used, evidence-based CAM methods. Concerning post-graduate education, it may be questioned whether the breakdown of CAM into several additional qualifications, as it is in Germany, is a good way for CAM. Concerns have been raised that this is contrary to the comprehensive approach of CAM. Probably, a better way would be to integrate (practical) CAM training into residency programmes. However, although more research and education will be needed, there no longer is a need to wait to use CAM: Cochrane database already includes over 5000 randomized, controlled trials on CAM.

### 4.6. Conclusion

Our study clearly demonstrates that CAM is highly valued by many FPs and is already making a substantial contribution to first-contact primary care in Germany. Considering the popularity CAM has in the public, integration of CAM is a chance for family medicine. However, FPs should be aware that patients expect not only to combine CAM and conventional medicine but to integrate CAM within a whole-system approach as “integrative medicine” [50]. “Integrative medicine” stands for a healing approach, allowing for the bio-psycho-socio-spiritual context of the individual patient, drawing on both, conventional medicine and CAM, on the basis of a trustful physician-patient relationship [54].

## Funding

BMBF (Federal Ministry of Education and Research) (FKZ: 01GK0514).

## Figures and Tables

**Table 1:** Basic characteristics of responding FPs.

	Our sample	Other samples
Gender (*n*)		
F	40%	39.5%^a^
M	59%	60.5%^a^
Age (years)	51.3 (min. 30, max. 71)	51.2^a^
Years of work in practice	15.2 (min. 1, max. 36)	Data not available
Structure of practice (*n*)		
Solo practice	525 (51%)	68%^b^
Location of practice (*n*)		
City	562 (55%)	50%^b^
Privately insured patients per practice (%)	12%	Data not available
Qualifications of FPs (*n*)		
Acupuncture	318 (31%)	Data not available^c^
Naturopathy	221 (21%)	8–18%^d^
Chiropractic	158 (15%)	8–20%^d^
Homeopathy	88 (9%)	3–8%^d^
Balneology	33 (3%)	1–3%^d^
Physical therapy	19 (2%)	2–4%^d^

^a^German Physician sample 2006; *Source*: National Association of SHI-Accredited Physicians [22].

^b^Sample of the Commonwealth-Fund-Survey 2006 [26, 27].

^c^Since acupuncture was newly accredited by the German federal medical current data are not available. It is estimated that around 30–40% of FPs had a training course in acupuncture.

^d^Exact data not available; numbers calculated from data of the German Physician sample 2006.

**Table 2:** Attitudes of FPs to statements about education, research and the provision of CAM.

	Disagree	Neutral	Agree
	(%)	(%)	(%)
FPs should have a *basic education* for the most important CAM disciplines	9	18	73
CAM should be provided *solely by physicians*	37	24	39
In specific cases I would *suggest* a patient to go to a *Heilpraktiker*	49	18	33
For the protection of patients there should be a quality control of *Heilpraktiker*	20	21	59
*Research* in CAM should be *increased*	15	17	68
